# Dietary supplementation with antibacterial peptide microcin J25 improved antioxidant capacity and intestinal health of pigeons

**DOI:** 10.3389/fvets.2025.1550776

**Published:** 2025-04-25

**Authors:** Heng Cao, Yinglin Lu, Xingyu Zhang, Fan Li, Ming Li, Jing Zhou, Huiting He, Qing Ma, Minli Yu

**Affiliations:** ^1^Department of Animal Genetics, Breeding and Reproduction, College of Animal Science and Technology, Nanjing Agricultural University, Nanjing, China; ^2^Department of Poultry Genetics and Breeding, Nanjing Institute of Animal Husbandry and Poultry Science, Nanjing, China

**Keywords:** antioxidant capacity, biochemical function, feed additive, microcin J25, pigeon

## Abstract

**Introduction:**

The present study aimed to evaluate the potential of antimicrobial peptide microcin J25 (MccJ25) as a feed additive for pigeons.

**Methods:**

A total of 28-day-old pigeons were allocated to four groups and fed a basal diet (CON) or a basal diet supplemented with 100, 200, or 300 mg/kg MccJ25 (ABP100, ABP200, and ABP300) for 8 weeks.

**Results:**

Dietary MccJ25 supplementation significantly improved survival rates in the ABP200 group compared to the control (*p* < 0.05). Serum analysis revealed that ABP200 and ABP300 groups exhibited increased levels of total protein (TP), globulin (GLB), immunoglobulin A (IgA), and immunoglobulin G (IgG), alongside reduced aspartate aminotransferase (AST), alanine aminotransferase (ALT), total cholesterol (TC), and triglyceride (TG) concentrations (*p* < 0.05). Furthermore, MccJ25 supplementation enhanced duodenal maltase and trypsin activity (*p* < 0.05) and upregulated intestinal and hepatic antioxidant capacity, as evidenced by elevated glutathione peroxidase (GSH-Px) and superoxide dismutase (SOD) activity (*p* < 0.05). Intestinal morphology improvements were observed in the jejunum and ileum, with increased villus height-to-crypt depth ratios (VH/CD) (*p* < 0.05). Additionally, ABP200 and ABP300 groups demonstrated significant upregulation of intestinal barrier-related genes occludin (*OCLN*), claudin 1 (*CLDN1*), zonula occluden protein 1 (*ZO1*), mucin 2 (*MUC2*), superoxide dismutase 1, 2 (*SOD1, 2*), and catalase (*CAT*) in duodenum (*p* < 0.05).

**Discussion:**

These findings indicate that MccJ25 supplementation improves systemic metabolism, enhances antioxidant defenses, strengthens intestinal barrier integrity, and ultimately promotes pigeon health and survival. This study supports the application of MccJ25 as a functional feed additive in poultry production.

## 1 Introduction

Antibacterial peptides (ABPs), small molecular peptides that are important to innate immune defense system (1, 2), have emerged as promising alternatives to antibiotics in livestock production due to their broad-spectrum antimicrobial activity and low risk of inducing resistance (3, 4). However, the potential effect of ABPs on pigeon production and their possibility of being used as a feed additive product still need to be studied. While studies in poultry (e.g., broilers) have demonstrated that AMPs enhance intestinal barrier function and modulate immune responses (1, 5), their application in pigeon production remains underexplored—a critical gap given the unique digestive physiology and commercial importance of pigeons in the global poultry industry.

Microcin J25 (MccJ25), a bacteriocin produced by Escherichia coli, has garnered attention for its unique stability and efficient antibacterial and anti-inflammatory properties (6). Recent advances highlight the interplay between AMPs and gut microbiota in mediating host health (7), and ABPs have been used as feed additives in livestock production (8, 9). Previous study showed that addition of ABP decreased the number of Escherichia coli in the ileum and cecum, which promoted intestinal health in broilers and then improved the production performance (10). Another study indicated that the addition of ABP to diets significantly reduced the number of coliforms in ileum and cecum and enhanced the humoral immune function, resulting in increased growth performance in weaned piglets (7, 11). It was also showed that dietary supplemented MccJ25 effectively improved performance, attenuated diarrhea and systematic inflammation, enhanced intestinal barrier function, and improved fecal microbiota composition of weaned pigs (12). In pigeons, however, the mechanisms by which AMPs influence gut microbiota composition, redox homeostasis, and systemic immunity remain unclear.

Pigeon meat industry has developed rapidly in recent years and has great market potential (13, 14). Pigeon squabs are first fed by crop milk secreted by their parents, and the digestive system of squabs is hypersensitive during the transition to self-feeding (15, 16). The weight of 28-day old King pigeons was 25.7 times that of the 1-day old, and then reaching their mature weight by day 28 (17). Our previous study found that ABP MccJ25 and improved the antioxidant capacity and production performance of pigeons, which is beneficial to the health of pigeon squabs (18).

This study aimed to evaluate the impact of MccJ25 supplementation on pigeon health and its potential as a feed additive. By integrating serum biochemistry, digestive enzyme profiling, and histopathological analyses, this work provides a comprehensive assessment of MccJ25′s potential as a sustainable feed additive, offering novel insights into AMP applications in niche poultry sectors.

## 2 Materials and methods

### 2.1 Ethics statement

This study was approved by the Laboratory Animal Welfare and Ethics Committee of Nanjing Agricultural University, China (Permit number: SYXK-2021-0014).

### 2.2 Animal experimental design, management and diet

A total of 360 28-day-old American Silver King pigeons (450 ± 10 g body weight) from a genetically homogeneous breeding line (Dongchen Pigeon Industry Co. Ltd., Nanjing, China) were randomly allocated into 4 dietary groups (n = 5 replicates/group). There were 18 pigeons for each replicate, which was divided into 6 cages with 3 pigeons per cage. Pigeons were raised in a ventilated three-layer ladder cage (45 × 40 × 40 cm^3^) with free access to feed and water. Pigeons were raised in a semi-open dovecote which was ventilated both mechanically and naturally, with an average temperature of 25°C-32°C, relative humidity 55–70% and natural light. The control group (CON) received a basal diet, and the experimental groups (ABP100, ABP200, and ABP300) were fed a basal diet supplemented with 100, 200, or 300 mg/kg MccJ25 (Zhongnong Yingtai Linzhou Biological Co. Ltd., purity: 94.7%) for 8 weeks. The composition and nutrient content of the pigeon diet are shown in Table 1.

**Table 1:** Basal diet nutrient levels and ingredients for pigeons.

**Items**	**Content**
**Ingredient composition (%)**	
Corn	42.55
Pea	25.53
Wheat	12.77
Sorghum	12.77
Green bean	6.38
Total	100.00
**Calculated nutrients**^a^ **(%)**	
Metabolizable energy (MJ/kg)	12.31
Crude protein	13.50
Crude fat	2.67
Calcium	0.08
Total phosphorus	0.31
**Analyzed nutrients (%)**	
Crude protein	13.43
Crude fat	2.94
Calcium	0.18
Total phosphorus	0.39
**Ingredients of grit meal**^b^ **(%)**	
Limestone	52.93
Shell meal	28.10
Yellow mud	14.05
Salt	1.41
Ferrous sulfate (monohydrate)	0.23
Premix^c^	3.28
Total	100.00

^a^Nutrition values and metabolizable energy values determined in pigeons were calculated from table of feed composition and nutritive values in China (31st edition, 2020).

^b^The grit meal was offered to pigeons consistently.

^c^The per kilogram of diet provided by the premix is as follows: vitamin A 5,000 IU, vitamin D_3_ 2,000 IU, vitamin E 500 IU; C_U_ (as copper sulfate) 15 mg, Fe (as ferrous sulfate) 100 mg, Mn (as manganese sulfate) 45 mg, Zn (as zinc sulfate) 90 mg.

### 2.3 Survival rate

At the end of trial, the number of died pigeons in each replicate was counted and the survival rate was calculated. The formula for survival rate for each repetition is as follows:


$$\begin{array}{r} \text{Survival Rate }(\%)=[(\text{Number of pigeons per repetition}\\ -Number\;of\;dead\;pigeons\;per\;repetition)\\ /Number\;of\;pigeons\;per\;repetition]\;\times \;100\% \end{array}$$

### 2.4 Sample collection

At 84 days of age, one pigeon from each replicate was randomly selected for left wing vein blood collection followed by cervical dislocation. Serum was separated by centrifugation of blood at 4,000 rpm for 15 min at 4°C and stored at −20°C. Duodenal and ileal contents were first collected aseptically, rapidly frozen and stored at −80°C. The intestines were re-moved, the intestinal contents were rinsed with phosphate buffer saline (PBS) and samples of duodenum, jejunum, ileum, jejunal mucosa, and liver were collected partly for intestinal morphology analysis and partly immediately cryopreserved at −80°C for further experiments.

### 2.5 Laboratory analysis

#### 2.5.1 Serum biochemical parameters

The serum biochemical parameters, including total protein (TP), aspartate ami-notransferase (AST), glucose (GLU), albumin (ALB), alanine aminotransferase (ALT), globulin (GLB), total cholesterol (TC), alkaline phosphatase (ALP) and triglyceride (TG) were measured spectrophotometrically using an automated system (CLS880 analyzer, Nanjing, China). The contents of immunoglobulin A (IgA), immunoglobulin G (IgG), and immunoglobulin M (IgM) in pigeon serum were assayed using commercial chicken elisa kits (Nanjing Aoqing Biotechnology Co., Ltd., CAT, ANG-E32004C, ANG-E32009C, and ANG-E32005C, Nanjing, China).

#### 2.5.2 Digestive enzyme activity

The contents of duodenum in pigeons were collected and were homogenized with ice-cold sodium chloride solution by using an Automatic Sample Rapid Grinding Machine. Then, the homogenization buffer was centrifuged at 3,500 rpm for 10 min at 4°C. The supernatant was used for assaying the enzyme activity of lipase (LPS), amylase (AMS), maltase, trypsin, and chymotrypsin in the duodenum contents of pigeons by using commercial kits (Nanjing Aoqing Biotechnology Co. Ltd., CAT, ZS3151, MZ4302, MZ4638, ZM4288, and ZM4292, respectively, Nanjing, China). The absorbance of each sample was spectrophotometrically measured using a spectrometer against a blank and then the activity was calculated according to the instructions provided by the manufacturer.

#### 2.5.3 Intestinal morphology

Approximately 1-cm intestinal segments (duodenum, jejunum and ileum) were collected and fixed in 4% paraformaldehyde for 24 h. Each sample was dehydrated, cleared and embedded in paraffin and cut at the thickness of 5 μm by using a microtome (LeicaBiosystems, Wetzlar, Germany). Then sections were stained with hematoxylin and eosin (H&E). Five fields of each section were randomly selected for image acquisition under a microscope (OlympusBX50, Tokyo, Japan). The villus height (VH), crypt depth (CD), and the ratio of villus height to crypt depth (V/C) were measured.

#### 2.5.4 Antioxidant indicators

The liver and serum samples were collected from pigeons on day 84 to test the activity of catalase (CAT), glutathione peroxidase (GSH-Px), superoxide dismutase (SOD), total antioxidant capacity (T-AOC), and the content of malonaldehyde (MDA). The level of SOD1 (Cu/Zn-SOD) and SOD2 (Mn-SOD) were measured respectively. Liver samples of approximately 1 g were homogenized with ice-cold sodium chloride solution according to the ratio of mass (g): volume (mL) = 1:9. Then, the homogenization buffer was centrifuged at 3,000 rpm for 10 min at 4°C. The supernatant was used for assaying the antioxidant indices by using commercial kits (Nanjing Aoqing Biotechnology Co. Ltd., CAT, YH1206, YH1267, YH1201, YH1248, and YH1218, respectively, Nanjing, China).

#### 2.5.5 Genes expression

Total RNA was extracted from intestinal, jejunal mucosa, and liver samples with Trizol, and cDNA was synthesized from the RNA using a cDNA synthesis kit (ABM, Richmond, CAN). The expression of target genes was measured using a BlasTaq 2 X qPCR MasterMix (ABM, Richmond, CAN) and a CFX Connect PCR Detection System (Bio-Rad, Hercules, CA, USA) using a reaction program of initial denaturation at 95°C for 3 min and then continued with 40 cycles of 95°C for 15 s and 60°C for 1 min. The primer sequence information of the genes is shown in Table 2. The relative mRNA expression of the target genes were calculated using the 2^−ΔΔCT^ method with β-actin as the control.

**Table 2:** Specific primers used for quantitative real-time PCR.

**Gene**	**Accession number**	**Primer sequences (5^′^ → 3^′^)**	**Product length**
*OCLN*	XM_005509325.2	F: GCCTCATCTGCTTCTTCGCTCAC R: GTCCACCACATTCTTCACCCACTC	134
*CLDN1*	XM_005513213.2	F: ACATCATGGTATGGCAACAGAGTGG R: ACAGGAGCAGCAGAGGAAGGC	150
*ZO-1*	XM_021299312.1	F: TGGGTGAGAAACGCTATGAG R: GCTTGTGATGTGCTGGGAGA	123
*MUC2*	XM_021296699.1	F: TGGCTCCACAGACAGGCAGAC R: TGGCTGACACATGAGGCACATTC	137
*SOD1*	XM_013370038.1	F: GCAGGGCATCATCCACTTCCAG R: ATCTCCGTCAGCCAAGCCATTG	82
*SOD2*	XM_013368727.2	F: AATGGAGGAGGAGAGCCTAAAGGAG R: AGCCTGATCCTTGAACACCAACTG	118
*CAT*	XM_005511042.2	F: CTGGAGAATCTGGCTCTGCTGATAC R: TGGATGAAGGACGGAAACAACAGTG	148
*ACTB*	XM_005504502.2	F: CCAGCCATGTATGTAGCCATCCAG R: AACACCATCACCAGAGTCCATCAC	90

OCLN, Occludin; CLDN1, Claudin 1; ZO-1, Zonula occluden protein 1; MUC2, Mucin 2; SOD1, Superoxide dismutase 1; SOD2, Superoxide dismutase 2; CAT, Catalase; ACTB, Actin beta.

### 2.6 Statistical analysis

Normality of data was confirmed via the Shapiro-Wilk test by using SPSS 20.0 software (SPSS Inc., Chicago, IL, USA). One-way ANOVA with Tukey multiple range test was applied for group comparisons. Survival rate was measured in replicates as the experimental unit, and the experimental unit for the other parameters was the individual bird. The statistical results were expressed as mean ± SEM, with significance set at *p* < 0.05. The graphs were drawn by using GraphPad Prism 6.0 software (San Diego, CA).

## 3 Results

### 3.1 Effect of ABP MccJ25 on survival rate in pigeons

Dietary MccJ25 supplementation significantly improved survival rates (p = 0.045; Table 3). From 28 to 84 days, the survival rate of all ABP groups was obviously increased compared with the control group, and ABP200 group showed the highest survival rate (Table 3).

**Table 3:** Effects of ABP MccJ25 on survival rate in pigeons from 28 to 84 days of age.

	**Treatment** ^**1**^				
**Item**	**CON**	**ABP100**	**ABP200**	**ABP300**	***P*** **-value** ^2^
Survival rate (%)	88.89 ± 1.76^b^	92.22 ± 1.36^ab^	95.55 ± 1.11^a^	93.33 ± 2.08^ab^	0.045

^1^CON: Basal diet; ABP100, ABP200, ABP300: Basal diet supplemented with 100 mg/kg 200 mg/kg, and 300 mg/kg antibacterial peptide MccJ25, respectively.

^2^p value was obtained by ANOVA; ^a, b^ The values in a row with different letters show significant differences (*p* < 0.05). Data were presented as mean ± SEM (n = 90).

### 3.2 Effect of ABP MccJ25 on biochemical and immunological parameters in pigeon serum

The impact of ABP treatment on the serum biochemical parameter of pigeons is presented in Table 4. Compared with the control group, ABP200 and ABP300 groups significantly increased the level of TP and GLB in pigeon serum (Table 4). ABP groups significantly reduced the level of AST and TG compared with the control group (Table 4), and the level of ALT and total TC were remarkably lower in the ABP200 and ABP300 groups than that of the control group (Table 4). But ALP and GLU showed no remarkable difference after the supplement of ABP (Table 4). The level of IgA and IgG in pigeon serum was significantly higher in all ABP groups than in the control group (Table 4). In addition, the content of IgM in pigeon serum was also increased in ABP groups, and it was significantly higher in ABP200 group than that of the control group (Table 4).

**Table 4:** Serum biochemical and immunological parameters of 84-day-old pigeons.

	**Treatment** ^**1**^				
**Items**	**CON**	**ABP100**	**ABP200**	**ABP300**	***p*** **value**^2^
TP (g/L)	26.84 ± 1.17^b^	27.20 ± 0.74^b^	32.34 ± 1.30^a^	33.60 ± 0.80^a^	< 0.001
ALB (g/L)	11.74 ± 0.31^b^	12.92 ± 0.52^ab^	13.12 ± 0.48^ab^	13.78 ± 0.65^a^	0.043
GLB (g/L)	15.10 ± 0.73^b^	14.48 ± 0.31^b^	19.22 ± 1.26^a^	19.82 ± 0.85^a^	< 0.001
AST (U/L)	124.26 ± 4.01^a^	112.22 ± 4.36^b^	113.30 ± 3.18^b^	107.86 ± 2.78^b^	0.035
ALT (U/L)	15.56 ± 0.48^a^	15.36 ± 0.69^ab^	13.66 ± 0.57^c^	13.92 ± 0.24^bc^	0.040
ALP (U/L)	446.76 ± 13.21	439.98 ± 20.2	438.72 ± 11.06	440.24 ± 8.85	0.976
GLU (mmol/L)	23.69 ± 0.73	24.72 ± 0.40	24.12 ± 0.48	26.65 ± 0.79	0.173
TC (mmol/L)	7.72 ± 0.48^a^	6.98 ± 0.24^ab^	6.62 ± 0.25^b^	6.71 ± 0.19^b^	0.034
TG (mmol/L)	2.08 ± 0.06^a^	1.82 ± 0.06^b^	1.68 ± 0.04^b^	1.66 ± 0.05^b^	< 0.001
IgA (ng/mL)	170.83 ± 6.07^c^	192.68 ± 7.84^b^	200.32 ± 4.37^b^	234.37 ± 5.66^a^	< 0.001
IgG (μg/mL)	190.86 ± 5.41^d^	243.09 ± 7.37^c^	275.88 ± 4.49^b^	298.81 ± 6.08^a^	< 0.001
IgM (μg/mL)	2.25 ± 0.05^b^	2.26 ± 0.08^b^	2.89 ± 0.11^a^	2.48 ± 0.08^b^	< 0.001

^1^CON: Basal diet; ABP100, ABP200, ABP300: Basal diet supplemented with 100 mg/kg 200 mg/kg, and 300 mg/kg antibacterial peptide MccJ25, respectively.

^2^P value was obtained by ANOVA; ^a − d^ The values in a row with different letters show significant differences (*p* < 0.05). Data were presented as mean ± SEM (n = 5).

TP, total protein; ALB, albumin; GLB, globulin; AST, aspartate aminotransferase; ALT, alanine aminotransferase; ALP, alkaline phosphatase; GLU, glucose; TC, total cholesterol; TG, triglyceride; IgA, immunoglobulin A; IgG, immunoglobulin G; IgM, immunoglobulin M.

### 3.3 Effect of ABP MccJ25 on the digestive enzyme activity in pigeon duodenum

Compared with the control group, the activity of maltase and trypsin in pigeon duodenal contents was significantly increased in ABP groups (Table 5), and chymotrypsin activity was also significantly increased in ABP200 and ABP300 groups (Table 5). In addition, AMS activity in the ABP200 group and LPS activity in the ABP300 group were significantly higher than control group (Table 5).

**Table 5:** Digestive enzyme activity in the duodenum of 84-day-old pigeons.

	**Treatment** ^**1**^				
**Items**	**CON**	**ABP100**	**ABP200**	**ABP300**	***p*** **value**^2^
LPS (U/g)	13.68 ± 0.49^b^	14.67 ± 0.64^b^	15.37 ± 0.52^b^	18.71 ± 0.64^a^	< 0.001
AMS (mg/min/mg)	0.66 ± 0.05^b^	0.72 ± 0.03^b^	1.09 ± 0.11^a^	0.75 ± 0.05^b^	0.001
Maltase (U/mg)	13.23 ± 0.58^c^	16.18 ± 0.53^b^	17.05 ± 0.39^b^	19.86 ± 1.16^a^	< 0.001
Trypsin (U/mg)	3.74 ± 0.15^c^	4.82 ± 0.29^b^	4.85 ± 0.32^b^	5.98 ± 0.40^a^	0.001
Chymotrypsin (U/mg)	6.11 ± 0.40^c^	6.76 ± 0.41^bc^	7.73 ± 0.29^ab^	8.57 ± 0.62^a^	0.007

^1^CON: Basal diet; ABP100, ABP200, ABP300: Basal diet supplemented with 100 mg/kg 200 mg/kg, and 300 mg/kg antibacterial peptide MccJ25, respectively.

^2^p value was obtained by ANOVA; ^a − c^ The values in a row with different letters show significant differences (*p* < 0.05). Data were presented as mean ± SEM (n = 5).

LPS, lipase; AMS, amylase.

### 3.4 Effect of ABP MccJ25 on the intestinal morphology and barrier function in pigeons

**Figure 1:**
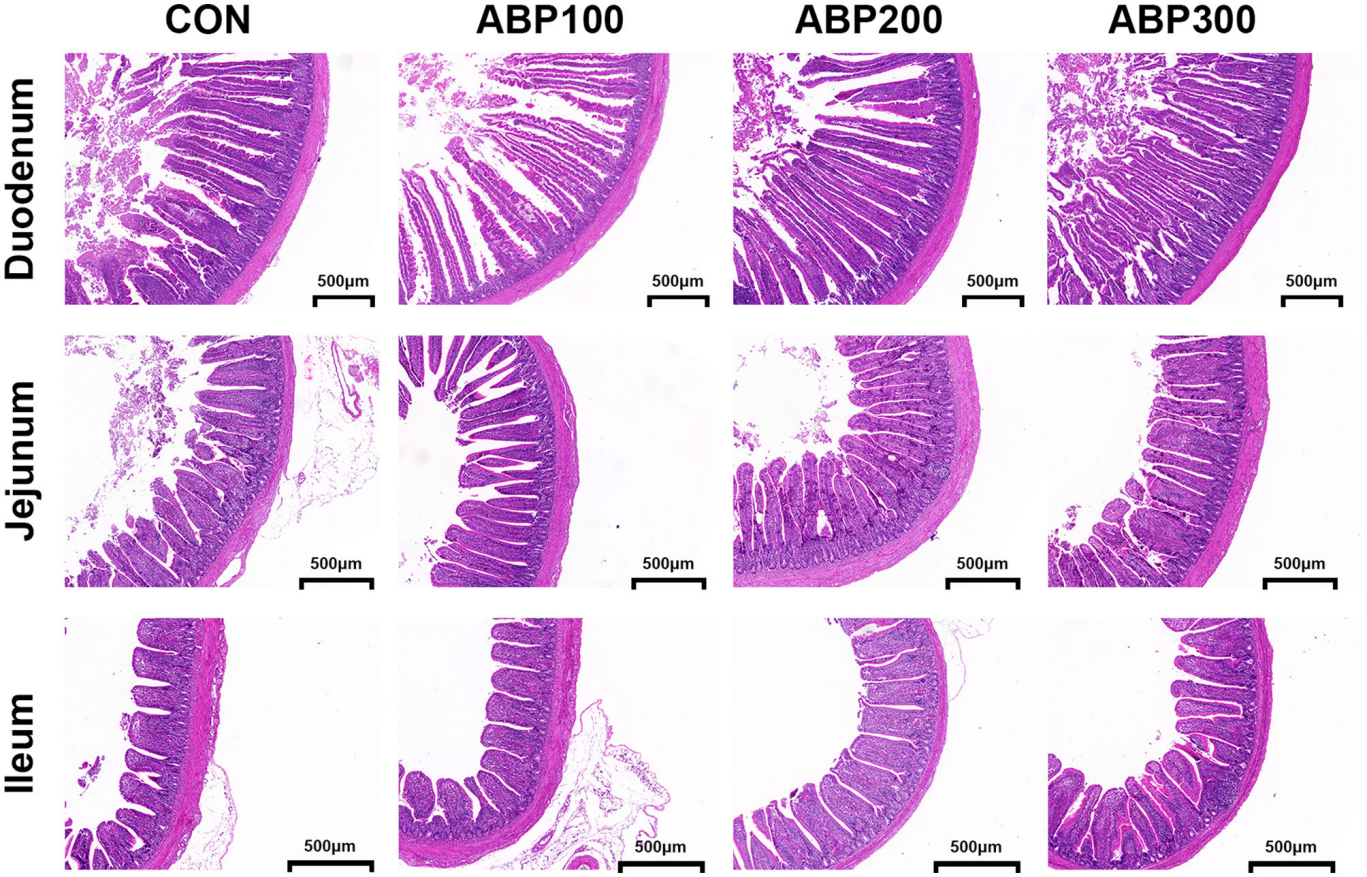
Intestinal morphology of 84-day-old pigeons. The morphology of intestinal villi was observed by hematoxylin and eosin (H&E) staining. Scale bar, 500 μm.

The intestinal villi of pigeons in ABP groups showed complete structure in morphology and high density compared with the control group (Figure 1). Villus height (VH) significantly increased by 15.4% (*p* < 0.001), V/C ratio by 12.7% in duodenum; VH (+10.8%) and V/C ratio (+11.3%) (*p* < 0.01) in jejunum; VH (+8.2%) and V/C ratio (+11.1%) (*p* < 0.05) in ileum by diet supplement with ABP200 (Table 6).

**Table 6:** Intestinal morphology of 84-day-old pigeons.

	**Treatment** ^**1**^				
**Items**	**CON**	**ABP100**	**ABP200**	**ABP300**	***p*** **value**^2^
**Duodenum**					
Villus height (μm)	1,308.80 ± 13.12^b^	1,336.02 ± 11.57^b^	1,510.77 ± 17.86^a^	1,487.35 ± 14.92^a^	< 0.001
Crypt depth (μm)	95.88 ± 0.72	98.17 ± 1.08	98.18 ± 1.08	97.62 ± 1.36	0.413
Villus height/Crypt depth	13.66 ± 0.20^b^	13.61 ± 0.12^b^	15.39 ± 0.12^a^	15.24 ± 0.14^a^	< 0.001
**Jejunum**					
Villus height (μm)	661.98 ± 11.67^b^	701.40 ± 12.26^a^	733.58 ± 11.54^a^	715.78 ± 13.83^a^	0.004
Crypt depth (μm)	94.92 ± 1.90	95.55 ± 2.21	94.48 ± 1.86	94.88 ± 1.70	0.815
Villus height/Crypt depth	6.97 ± 0.08^b^	7.34 ± 0.11^a^	7.76 ± 0.11^a^	7.54 ± 0.12^ab^	< 0.001
**Ileum**					
Villus height (μm)	349.82 ± 5.21^b^	362.27 ± 6.94^ab^	378.37 ± 7.18^a^	377.92 ± 7.39^a^	0.019
Crypt depth (μm)	80.65 ± 1.26^ab^	80.27 ± 1.27^ab^	78.50 ± 0.68^b^	82.17 ± 0.60^a^	0.038
Villus height/Crypt depth	4.34 ± 0.08^c^	4.51 ± 0.12^b^	4.82 ± 0.14^a^	4.60 ± 0.17^b^	< 0.001

^1^CON: Basal diet; ABP100, ABP200, ABP300: Basal diet supplemented with 100 mg/kg 200 mg/kg, and 300 mg/kg antibacterial peptide MccJ25, respectively.

^2^p value was obtained by ANOVA; ^a−*c*^ The values in a row with different letters show significant differences (*p* < 0.05). Data were presented as mean ± SEM (n = 5).

**Figure 2:**
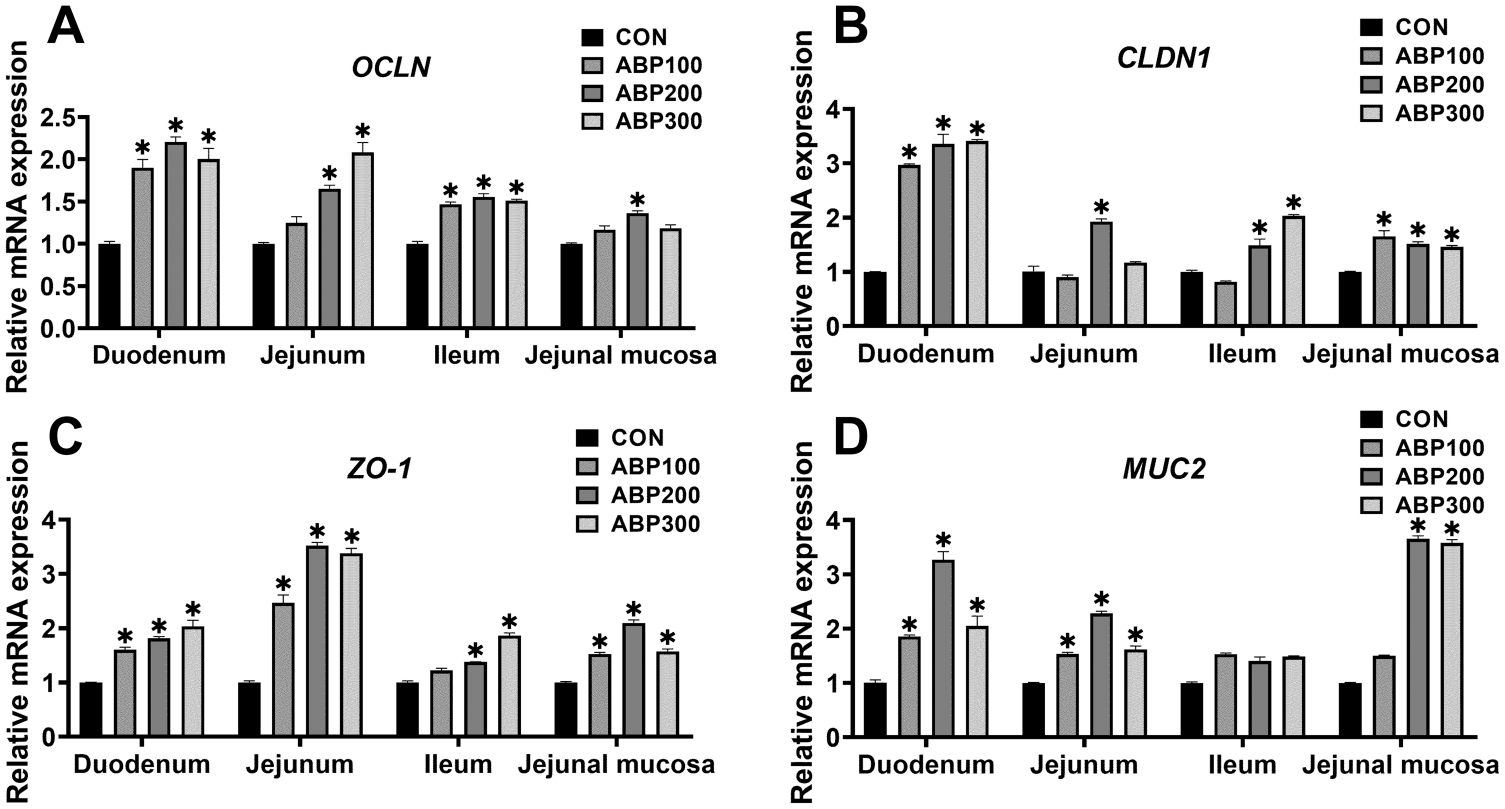
Relative mRNA expression of intestinal barrier-related genes in 84-day-old pigeons. The mRNA expression of tight junction genes *OCLN* **(A)**, *CLDN1* **(B)**, *ZO-1* **(C)** and *MUC2* **(D)** was measurted by qRT-PCR. *OCLN*, Occludin; *CLDN1*, Claudin 1; *ZO-1*, Zonula occluden protein 1; *MUC2*, Mucin 2. Data are presented as mean ± SEM. ns, *p* > 0.05; *p < 0.05 vs. CON.

ABP groups significantly up-regulated the mRNA expression level of the OCLN, CLDN1, ZO-1 and MUC2 in pigeon duodenum compared with control group (Figure 2). In pigeon jejunum, ABP groups obviously up-regulated the mRNA expression level of the ZO-1 and MUC2 compared with control group, and ABP200 and ABP300 groups significantly increased the mRNA expression of OCLN (Figure 2). In pigeon ileum, compared with control group, the mRNA expression of the OCLN, CLDN1 and ZO-1 was significantly increased in the ABP200 and ABP300 groups (Figure 2). In pigeon jejunal mucosa, compared with the control group, ABP200 and ABP300 groups significantly up-regulated the mRNA expression level of the CLDN1, ZO-1 and MUC2 (Figure 2).

### 3.5 Effect of ABP MccJ25 on the antioxidant capacity in pigeons

In pigeon serum, the activity of T-AOC and GSH-PX was remarkably increased and MDA content was dramatically decreased in ABP200 and ABP300 groups compared with the control group (Table 7). The activity of SOD in ABP200 group was significantly higher than control group (Table 7). In pigeon liver, ABP200 and ABP300 groups notably enhanced the activity of GSH-PX, SOD and CAT in pigeons compared with the control group, but the content of MDA in ABP200 group was significantly lower than the control group (Table 7).

**Table 7:** Antioxidative parameters in the serum and liver of 84-day-old pigeons.

	**Treatment** ^**1**^				
**Items**	**CON**	**ABP100**	**ABP200**	**ABP300**	***p*** **value**^2^
**Serum**					
T-AOC (U/mL)	49.96 ± 2.05^b^	49.75 ± 2.55^b^	58.43 ± 2.00^a^	57.70 ± 1.33^a^	0.009
MDA (nmol/mL)	0.90 ± 0.03^a^	0.88 ± 0.04^a^	0.77 ± 0.01^b^	0.78 ± 0.03^b^	0.015
GSH-Px (nmol/min/mL)	46.85 ± 2.35^c^	51.33 ± 2.49^bc^	60.89 ± 2.5^a^	55.10 ± 1.81^ab^	0.004
SOD (U/mL)	12.17 ± 1.14^b^	12.11 ± 1.00^b^	17.03 ± 1.10^a^	15.01 ± 0.89^ab^	0.010
**Liver**					
T-AOC (U/mL)	17.79 ± 0.41^b^	18.49 ± 1.17^b^	19.30 ± 1.23^b^	25.11 ± 0.78^a^	< 0.001
MDA (nmol/mL)	1.28 ± 0.01^a^	1.17 ± 0.05^ab^	1.09 ± 0.03^b^	1.16 ± 0.06^ab^	0.049
GSH-Px (nmol/min/mL)	21.41 ± 1.49^c^	21.01 ± 1.33^c^	26.39 ± 1.61^b^	33.24 ± 2.06^a^	< 0.001
SOD (U/mL)	134.23 ± 6.79^c^	148.16 ± 9.73^bc^	169.42 ± 6.15^b^	230.20 ± 7.31^a^	< 0.001
CAT(μmol/min/mL)	16.08 ± 0.55^c^	16.16 ± 0.45^c^	22.11 ± 0.81^b^	24.55 ± 0.74^a^	< 0.001

^1^CON: Basal diet; ABP100, ABP200, ABP300: Basal diet supplemented with 100 mg/kg 200 mg/kg, and 300 mg/kg antibacterial peptide MccJ25, respectively.

^2^p value was obtained by ANOVA; ^a−*c*^ The values in a row with different letters show significant differences (*p* < 0.05). Data were presented as mean ± SEM (n = 5).

T-AOC, total antioxidant capacity; MDA, malonaldehyde; GSH-Px, glutathione peroxidase; SOD, superoxide dismutase; CAT, catalase.

**Figure 3:**
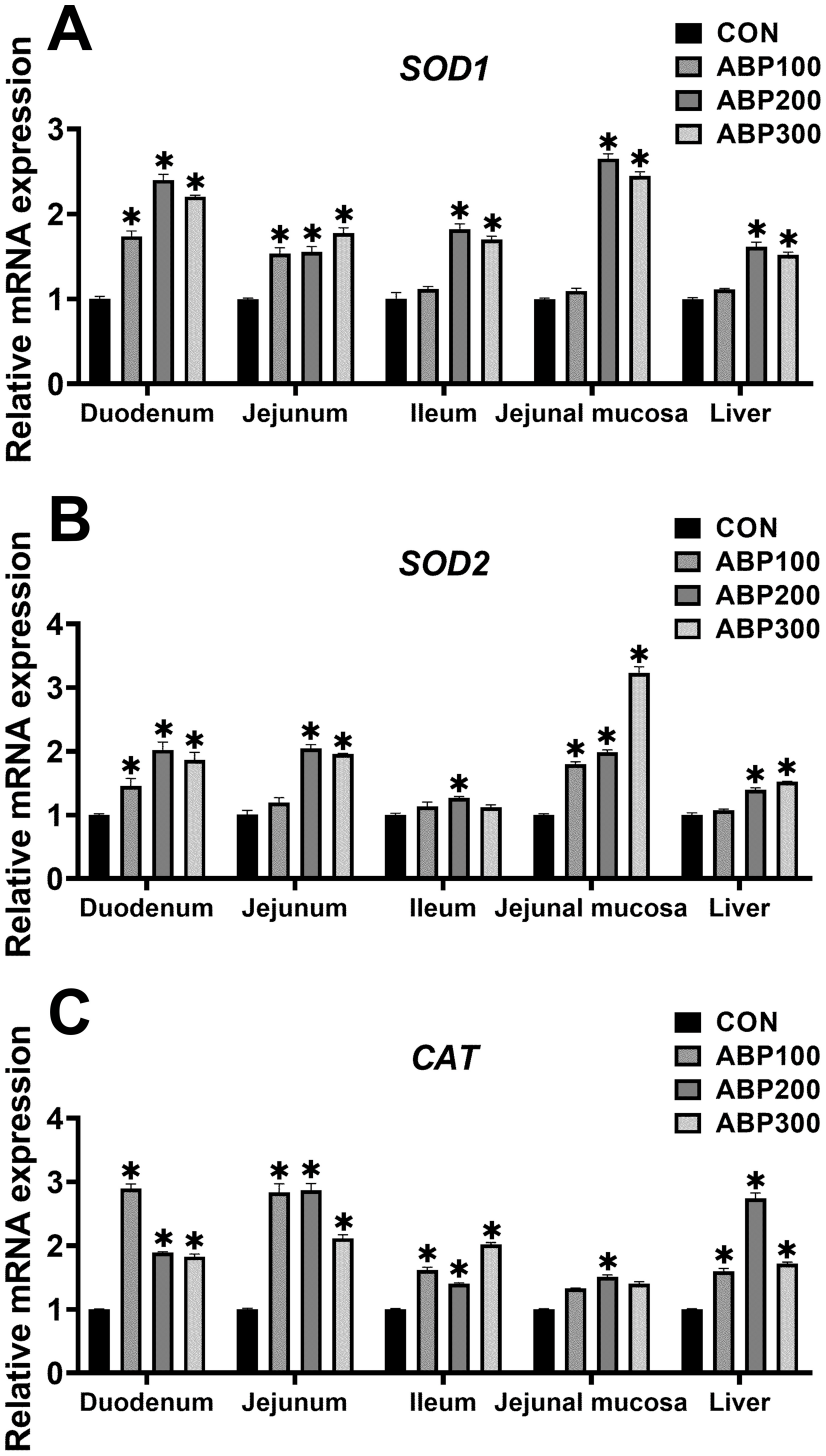
Relative mRNA expression of antioxidant relative genes in intestinal and liver in 84-day-old pigeons. The mRNA expression of the antioxidant genes *SOD1* **(A)**, *SOD2* **(B)**, and *CAT* **(C)**. *SOD1*, Superoxide dismutase 1; *SOD2*, Superoxide dismutase 2; *CAT*, Catalase. Data are presented as mean ± SEM (*n* = 5). ns, *p* > 0.05; *p < 0.05 vs.CON.

Compared with the control group, the ABP200 and ABP300 groups significantly up-regulated the mRNA expression level of the SOD1, SOD2 and CAT in pigeon duo-denum, jejunum and liver (Figure 3). The mRNA expression of the SOD1 and CAT was significantly increased in the ABP200 and ABP300 groups in pigeon ileum compared with the control group (Figure 3). In pigeon jejunal mucosa, ABP200 and ABP300 groups significantly increased the mRNA expression of SOD1 and SOD2 compared with the control group, and the mRNA expression of CAT was also significantly increased in ABP200 group (Figure 3).

## 4 Discussion

Antimicrobial peptides (AMPs) represent a promising alternative to antibiotics in livestock production, with demonstrated efficacy in enhancing gut health, immune function, and oxidative homeostasis across species (19–21). Pigeons are susceptible to bacterial infections in the early stages of growth, leading to a low survival rate, therefore, this study aimed to investigate the potential application of ABP MccJ25 in young pigeons. The percent study revealed that MccJ25 supplementation at 200 mg/kg exerted significant effects on intestinal barrier reinforcement, redox balance modulation, and humoral immunity enhancement, then improved survival rates in pigeons.

The improved intestinal morphology (villus height, V/C ratio) and upregulated tight junction proteins (OCLN, CLDN1, ZO-1) observed here are consistent with AMP-mediated gut protection mechanisms reported in poultry (10, 22, 23). It has been shown that the addition of ABPs in the diet remarkably increased the VH of jejunum, improved the V/C of duodenum and ileum, and significantly increased the expression of the intestinal tight junction protein genes (*ZO-1, CLDN3*, and *MUC2*) in broilers (24). Notably, the enhanced MUC2 expression suggests MccJ25 promotes mucin secretion, a critical component of the mucosal barrier against pathogens (25). The improved intestinal morphology and barrier function may be related to the intestinal flora, and ABP Plectasin significantly reduced the number of *Escherichia coli* (*E. coli*) in the ileum of yellow-feathered chickens, improved intestinal morphology, and significantly increased the expression of barrier genes *ZO-1* and *CLDN3* in the ileum (26). Similarly, this study found that ABP MccJ25 significantly increased the VH, decreased the V/C of small intestine, improved the intestinal morphology in pigeons and was conducive to intestinal absorption of nutrients, which may be the reason for the increase of its duodenal digestive enzyme activity. Recent studies highlight that polysaccharide-induced microbiota modulation alleviates oxidative organ damage by restoring microbial balance (27), while a study identified host-microbe interactions as key determinants of intestinal barrier integrity in swine (28). Although our study did not directly profile gut microbiota, the improved digestive enzyme activity (maltase, trypsin) and reduced systemic inflammation (lower AST/ALT) imply MccJ25 may exert prebiotic-like effects by suppressing pathogenic bacteria (e.g., E. coli) while favoring commensal species—a mechanism previously observed with plectasin in poultry (26). Future metagenomic analyses, as proposed by Huang et al. (28), could clarify MccJ25′s microbiota-specific impacts in pigeons.

Serum biochemical indicators indicate the nutritional metabolism and health status of the animal (29–31). Previous study showed that serum biochemical indicators were improved by the addition of ABPs in broilers (32). Similar results was obtained in this study that the addition ABP MccJ25 significantly increased the level of TP, ALB and GLB in pigeon serum, indicating that protein metabolism was vigorous and the degree of protein digestion and utilization was improved. It has been shown that ABPs significantly reduced TC content in the serum of weaned piglets (33), which potentially via AMP-mediated lipid regulation (34). Our observation of reduced serum TC/TG levels in MccJ25-supplemented pigeons further supports this result. It has been shown that ABPs elevated the activity of intestinal AMS, LPS and trypsin in broilers (10). This study showed that ABP MccJ25 increased the activity of duodenal AMS, maltase and LPS in pigeons and promoted the absorption and utilization of nutrients in the duodenum.

Serum AST and ALT reflect liver function or liver damage (35). In our study, ABP MccJ25 showed a significant decrease in the level of AST and ALT in pigeon serum, suggesting a protective effect of ABP MccJ25 on the liver. Immunoglobulins (IgG, IgA, IgM) are important components of the humoral immune system (10, 36). Previous studies found that ABP Plectasin significantly increased the levels of IgG and IgM in serum of broilers and weaned piglets (11). The use of ABP pratt and full-tide promotes the development of immune organs such as the bursa and thymus in broilers, and has the effect of preventing diseases and reducing mortality in broilers (24). Additionally, the elevated immunoglobulins (IgA, IgG, IgM) corroborate findings by Chen et al. (37). The present results were similar to the previous results, where ABP MccJ25 significantly increased the levels of IgA, IgG and IgM in pigeon serum, enhanced their humoral immune function, and increased the survival rate of the pigeons. This result corroborated the immune function of ABPs (2, 4, 20).

When the oxidative balance of the body is broken, it will cause oxidative stress in the organism, resulting in cell and tissue damage, which leads to a variety of diseases (38). SOD, GSH-PX, and CAT synergistically eliminate free radicals and peroxides to reduce oxidative damage (13). MDA is lipid peroxide produced by the metabolism of free radicals in the body (39), and its content is inversely proportional to the health status of the body. Consistent with previous studies, the results showed that ABP groups led to significant increases in T-AOC, GSH-Px, SOD, and CAT levels, while reducing MDA content in both pigeon serum and liver, and enhanced the oxidative defense system and scavenging capacity of the body against oxidative factors. It has been shown that ABPs improved the intestinal flora and showed resistance to bacterial infections, and also significantly increased the expression of intestinal antioxidant genes *SOD1* and *CAT* to enhance the antioxidant capacity of broiler chickens (20). It was also found in this study, suggesting the enhanced antioxidant capacity of ABPs in pigeons. While the potential influence of confounding factors such as genetic variability and environmental conditions on experimental outcomes was existed, future studies will delineate microbiome differences or epigenetic modifications induced by these factors. Despite these limitations, our study provided robust support for the causality inferred between MccJ25 supplementation and the observed phenotypic improvements.

The survival rate improvement (6.7% increase in ABP200 vs. CON) holds significant economic potential for pigeon farming. ABP MccJ25 indirect health promotion via gut-liver axis modulation. Notably, the absence of adverse effects at 300 mg/kg contrasts with reports of high-dose AMP toxicity in mice (40, 41), suggesting pigeons may tolerate higher doses for therapeutic applications. However, pigeon-specific microbiota interactions, long-term safety, and comparative efficacy with structurally distinct AMPs (e.g., cLFchimera) warrant further investigation. Future studies should integrate multi-omics approaches to decode host-microbe crosstalk and clarify structure-function relationships in avian species.

## 5 Conclusion

The present study demonstrated that dietary supplementation with ABP MccJ25 improved serum biochemical indices, elevated intestinal digestive enzyme activity and strengthened systemic antioxidant capacity and ameliorated intestinal barrier integrity, thereby improved survival rates in pigeons. These results collectively highlight the roles of ABP MccJ25 in promoting intestinal health. Based on its dose-dependent efficacy, the inclusion of 200 mg/kg MccJ25 in diets is recommended as a sustainable strategy for improving productivity in pigeons. This investigation provides both theoretical and practical foundations for adopting ABP MccJ25 additives as alternatives in the pigeon industry.

## Data Availability

The original contributions presented in the study are included in the article/supplementary material, further inquiries can be directed to the corresponding author.
